# Population Pharmacokinetic/Pharmacodynamic Model-Guided Dosing Optimization of a Novel Sedative HR7056 in Chinese Healthy Subjects

**DOI:** 10.3389/fphar.2018.01316

**Published:** 2018-11-19

**Authors:** Ying Zhou, Pei Hu, Yuguang Huang, Nuoer Sang, Kaicheng Song, Hongyun Wang, Jinhua Wen, Ji Jiang, Xia Chen

**Affiliations:** ^1^Clinical Pharmacology Research Center, Peking Union Medical College Hospital, Peking Union Medical College and Chinese Academy of Medical Sciences, Beijing, China; ^2^Department of Pharmacy, The First Affiliated Hospital of Nanchang University, Nanchang, China; ^3^Department of Anesthesiology, Peking Union Medical College Hospital, Peking Union Medical College and Chinese Academy of Medical Sciences, Beijing, China; ^4^Clinical Trial Center, China National Clinical Research Center for Neurological Diseases, Beijing Tiantan Hospital, Capital Medical University, Beijing, China

**Keywords:** population pharmacodynamics, population pharmacokinetics, sedation, modeling, benzodiazepine

## Abstract

HR7056 is a new benzodiazepine, showing more faster acting onset and recovery than currently available short-acting sedatives. To avoid inadequate anesthesia and predict return of cognition, allowing for immediate neurological evaluation, HR7056 pharmacokinetics and pharmacodynamics were characterized in Chinese healthy subjects. We report on modeling of the data and simulations of dosage regimens for future study. Up to 63 subjects were evaluated, using Bispectral Index (BIS) and Modified Observer's Assessment of Alertness/Sedation (MOAA/S) as pharmacodynamics endpoints. A three-compartment model best described HR7056 pharmacokinetics. Total clearance was 1.49 L min^−1^, central volume was 2.1 L, inter-compartmental clearances were 0.96 and 0.27 L min^−1^, respectively. The population mean pharmacodynamic parameters were as follows: BIS, E_0_: 95.3; IC_50_: 503 ng mL^−1^; γ: 1.5; k_e0_: 0.0855 min^−1^; I_max_: 47.9 and MOAA/S, IC_50_: 436 ng mL^−1^; γ: 1.5; k_e0_: 0.05 min^−1^; I_max_: 27.9. The model simulation will enable maintenance doses to be given more accurately for future study.

**Clinical Trial Registration**: identifier: NCT01970072

## Introduction

Remimazolam, which is synthesized to undergo rapid hydrolysis by non-specific tissue esterases in the body to its pharmacologically inactive carboxylic acid metabolite, is a new benzodiazepine class of sedative drugs (Kilpatrick et al., 2007). Preclinical and clinical studies showed that remimazolam possessed a faster onset, a shorter duration of sedative action, and a more rapid recovery than currently available short-acting sedatives (Rogers and McDowell, 2010; Upton et al., 2010). Herein, owing to its fast-acting onset and recovery and organ-independent metabolism, remimazolam seemingly have potential advantages when used as an intravenous sedative agent for the induction and maintenance of anesthesia.

Remimazolam Tosilate (HR7056, Figure S1) was approved by China Food and Drug Administration as an investigational new drug for the potential use in minor operations anesthesia in 2013 and is currently being evaluated in phase III trials. To avoid inadequate anesthesia and predict return of cognition, allowing for immediate neurological evaluation, it is necessary to develop pharmacokinetic and pharmacodynamic models of HR7056 and simulate the effects of varied courses of therapy so as to guide dosing optimization of the new drug. We report on modeling of the data and simulations of dosage regimens, which will enable maintenance doses to be given more accurately for future study. To our knowledge, this is the first report of the pharmacokinetics and pharmacodynamics of HR7056 in Chinese healthy population. Our study provides valuable information for the development and application of HR7056 in clinic.

## Methods

### Clinical study and subjects

The clinical study which is in full compliance with the principles of the Declaration of Helsinki and Good Clinical Practice Guidelines, was conducted at the Clinical Pharmacology Research Center of the Peking Union Medical College Hospital. The trial was registered prior to patient enrollment at ClinicalTrials.gov (NCT01970072, Principal investigator: Bei Hu, Professor, Date of registration: October 25, 2013). This was a single-center, double-blinded, randomized, single ascending-dose study of HR7056 administered as a 1-min IV injection, compared with midazolam. Up to 63 Chinese healthy subjects who received HR7056 were planned for enrollment in up to 11 cohorts (doses ranging from 0.01 to 0.45 mg kg^−1^). Sixteen subjects received midazolam (6, 0.075 mg kg^−1^; 10, 0.12 mg kg^−1^). Subjects eligible to take part in this study were healthy males or females, ages from 18 to 55 years inclusive, weighing between 50 and 100 kg, with a body mass index (BMI) of 18–26 kg m^−2^. The study was approved by the Ethics Committee of Peking Union Medical College Hospital, and all subjects signed the Informed Consent Form before the study. This manuscript adheres to the applicable CONSORT guidelines.

During the study, sedation was measured by Bispectral Index (BIS) (Bower et al., 2000) monitoring and Modified Observer's Assessment of Alertness/Sedation (MOAA/S) (Chernik et al., 1990) score assessments pre-dose and at 1, 2, 3, 4, 6, 8, 10, 12, 15, 20, 25, 30, 35, 40, 45, 50, 60, and 120 min after the start of the infusion. The study would stop if loss of consciousness (MOAA/S < 2) for ≥5 m was observed in >50% of subjects in a single cohort. Table S1 shows the MOAA/S scoring standard used.

Arterial plasma samples were collected at pre-dose and 1, 2, 3, 4, 6, 8, 10, 12, 15, 20, 30, 45, 60, 120, 180, 240, 360, and 480 min post-dose. All samples were stored at −70°C until analysis. Plasma concentrations of HR7056 were measured using ultra-performance liquid chromatography with tandem mass spectrometric detection as described in our previous report (Zhou et al., 2015). The method was fully validated over the concentration range of 0.5–1,000 ng mL^−1^. The lower limit of quantification (LLOQ) was 0.5 ng mL^−1^. Inter- and intra-batch precision and the accuracy were well within the acceptable range. Selectivity, matrix effect and stability were also investigated.

### Population pharmacokinetic modeling

A population approach was applied to model the pharmacokinetic/pharmacodynamic behavior of HR7056. The population pharmacokinetic analysis was performed using a non-linear, mixed-effect modeling program (Phoenix NLME, version 1.2). First-order conditional estimation with interaction method (FOCE-I) was used. A three-compartment model was developed and these were expressed as clearances and volumes, of which the inter-subject variability was modeled assuming log-normal distributions:

$$\begin{array}{l} \theta_{i}=\theta_{TV}\cdot e^{\eta_{i}} \end{array}$$

where θ_*i*_ is the parameter value in the *i*th patient, θ_*TV*_is the typical value of the parameter in the population, and η_*i*_ is a random variable in the *i*th patient with a mean of 0 and a variance of ω^2^. Inter-individual variability is reported as ω, the SD of η in the log domain, which is approximately the coefficient of variation in the standard domain. A proportional error model was used for the residual random effects.

The choice of the final structural model was determined by the objective function (OFV). Body weight, height, age, and gender were examined in turn as possible covariates. The OFV was the criterion used to determine whether the introduction of a particular covariate was statistically significant, of which reductions of 3.84, 6.63, and 10.83 were statistically significant at the 0.05, 0.01, and 0.001 levels, respectively.

### Population pharmacodynamic modeling

A “link” model was used to relate plasma concentrations of HR7056 to levels at the tissue “effect” site and a sigmoid inhibitory effect model described the relationship between HR7056 effect-site concentration (*C*_*e*_) and drug effect (BIS and MOAA/S scores) (Coppens et al., 2011):

$$\begin{array}{l} \frac{dC_{e}}{d_{t}}=k_{eo}\cdot \left(C_{p}-C_{e}\right)\;and\;E=E_{0}-\frac{I_{Max}\cdot {C_{e}}^{\gamma }}{IC_{50}^{\gamma }+C_{e}^{\gamma }} \end{array}$$

where *k*_*eo*_ is the equilibration rate constant between the arterial plasma and effect compartments, *C*_*p*_ and *C*_*e*_ are the arterial plasma concentration and effect-site concentration of HR7056, respectively. *E*_0_ is the baseline value of drug effect (BIS), *I*_*Max*_ is the maximum possible reduction in drug effect, *IC*_50_ is the concentration of drug that causes the value of drug effect to decrease to halfway between *E*_0_ and (*E*_0_-*I*_*Max*_), and γ (the Hill coefficient) is a coefficient that describes the shape of the sigmoidal curve.

Because the MOAA/S scale is categorical, ordered categorical models were fitted to the data, using NONMEM (version 7.2), but with a logistic function and the conditional Laplacian method of estimation. Studies (Sandler and Sparks, 2000; Zhong et al., 2005; Barakat et al., 2007; Balci et al., 2011; Colin et al., 2017) have shown that there is a good positive correlation between MOAA/S score and BIS. Therefore, we assumed that MOAA/S score related effect-site concentration was the same as that of BIS. The cumulative logit for each category was calculated and hence the cumulative probabilities that the MOAA/S score was less than or equal to that category.

$$\begin{array}{l} Logit\left(x\right)=Baseline\left(x\right)+\frac{I_{Max}\cdot C_{e}^{\gamma }}{IC_{50}^{\gamma }+C_{e}^{\gamma }}+\eta \end{array}$$

where “*x*” represents a particular MOAA/S score and *Baseline(x)* is the probability of achieving that score in the absence of the drug, *I*_*Max*_ is the maximal achievable probability, and η represents any inter-subject variability. The logits were converted into cumulative probabilities P(*x*) by means of the logistic transformation:

$$\begin{array}{l} P\left(x\right)=\frac{1}{1+e^{-Logit\left(x\right)}} \end{array}$$

Subsequently, the probabilities for each category were calculated by subtraction from the cumulative probabilities, with the probability to observe a MOAA/S score ≤ 5 being 1.

### Parameter estimation and model evaluation

The population pharmacodynamic analysis of the BIS data was performed using Phoenix NLME (version 1.2), with an additive residual error model. For the categorical MOAA/S data, the Laplacian approximation to the likelihood was used by NONMEM (version 7.2).

During model building, the goodness of fit (GOF) of the different models was compared using the Akaike information criterion (AIC). In the meanwhile, GOF was graphically evaluated by inspecting plots of the individual or population predicted vs. observed responses, and plots of the conditionally weighted residuals (CWRES) vs. population predictions and time. Finally, models were validated internally using prediction-corrected visual predictive checks (pcVPC) (Bergstrand et al., 2011).

### Statistical analysis

All model parameters are shown in the manner of typical values with relative standard errors (RSE) and 95% confidence interval (CI) derived from log-likelihood profiling (Venzon and Moolgavkar, 1988).

### Simulations

In order to predict the target effect of the induction and maintenance of anesthesia and guide dosing optimization for future study, Monte-Carlo simulations of the pharmacokinetics and pharmacodynamics of HR7056 with different dosage regimens were performed based on the final model. Combinations of 0.4 mg kg^−1^ min^−1^ (infusion within 1 min) initial loading dose with 1.0, 1.5, 3.0, and 6.0 mg kg^−1^ hr^−1^ (infusion for 2 h) maintenance dose regimens were examined, respectively. The optimal dosing regimen was selected on the basis of target effect which was defined as maintenance of a MOAA/S score < 2 for at least 1.5 h. One thousand virtual subjects were simulated each time, and the pharmacokinetic and pharmacodynamic values for the 1,000 subjects were simulated using the parameters from the final models, of which the variability were randomly sampled from the log-normal distributions obtained from the modeling.

## Results

**Figure 1:**
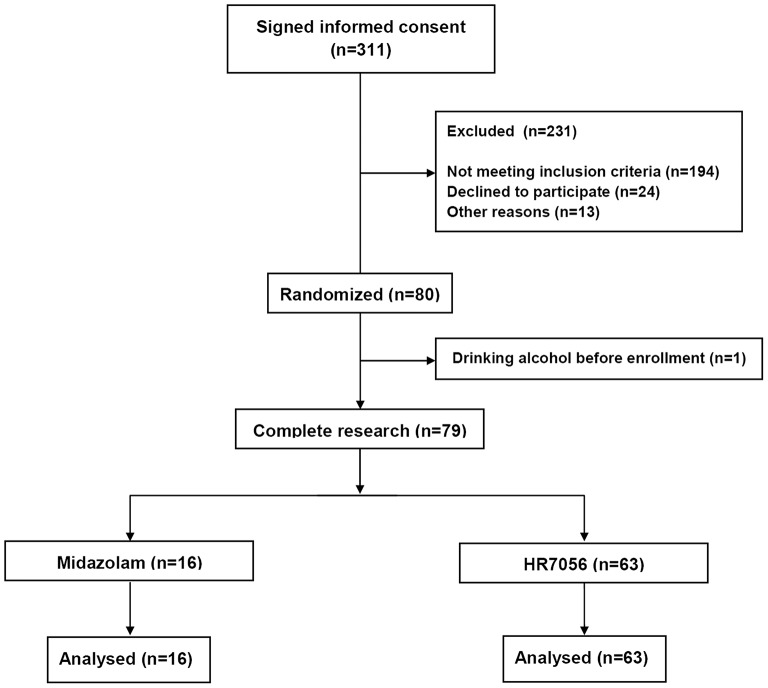
Study flow diagram.

In the screening period, 194 subjects did not meet the inclusion criteria. Common causes of failed screening were abnormal laboratory, abnormal physical examination, positive smoking test, and inappropriate age or BMI (Figure 1).

Data from all included 63 subjects were used in the analysis. In total, 1,197 arterial plasma samples, 1,197 BIS value, and 1,197 MOAA/S scores were, respectively obtained and used for the modeling. In brief, 51 males and 12 females were included. Their demographics were (median [min–max]): age, 27 y [18–44 y]; weight, 63.8 kg [52.8–83.8 kg]; height, 169 cm [156–184 cm].

### Population pharmacokinetic analysis of HR7056

Based on the AIC, a three-compartment model fit the data well. Covariate analysis of the possible effects of body weight, height, age, and gender was examined for each pharmacokinetic parameter. No covariate effects were statistically significant at the 0.01 level (Δ*OFV* = −6.63). The typical values, intra-individual and residual variability for the estimated population model, are shown in Table 1. The performance of the final model of HR7056 was good with relative standard errors of the population mean pharmacokinetic parameters ranging from 1.9 to 4.1%. Inter-individual variability ranged from 11.5 to 25.3% and residual variability was 13.8%.

**Table 1:** Summary of final population pharmacokinetic model of HR7056.

**Parameter**	**Typical value (RSE%)**	**95% CI**	**IIV% (RSE%)**
Central (arterial) volume (L)	2.11 (4.0)	(1.94–2.27)	14.0(5.6)
Elimination clearance (L min^−1^)	1.49 (1.9)	(1.44–1.55)	11.5 (2.3)
Peripheral volume V_2_ (L)	10.5 (3.9)	(9.73–11.3)	12.2 (5.2)
Inter-compartmental clearance Cl_2_ (L min^−1^)	0.96 (3.7)	(0.89–1.03)	13.3 (4.5)
Peripheral volume V_3_ (L)	22.7 (4.1)	(20.9–24.6)	25.3 (5.8)
Inter-compartmental clearance Cl_3_ (L min^−1^)	0.27 (4.0)	(0.250–0.295)	18.7 (7.8)
σ	0.138 (2.9)	(0.130–0.145)	–

*–, Not applicable; 95% CI, 95% confidence interval; IIV, Inter-individual variability; RSE%, Percentage relative standard error; σ, Residual variability*.

**Figure 2:**
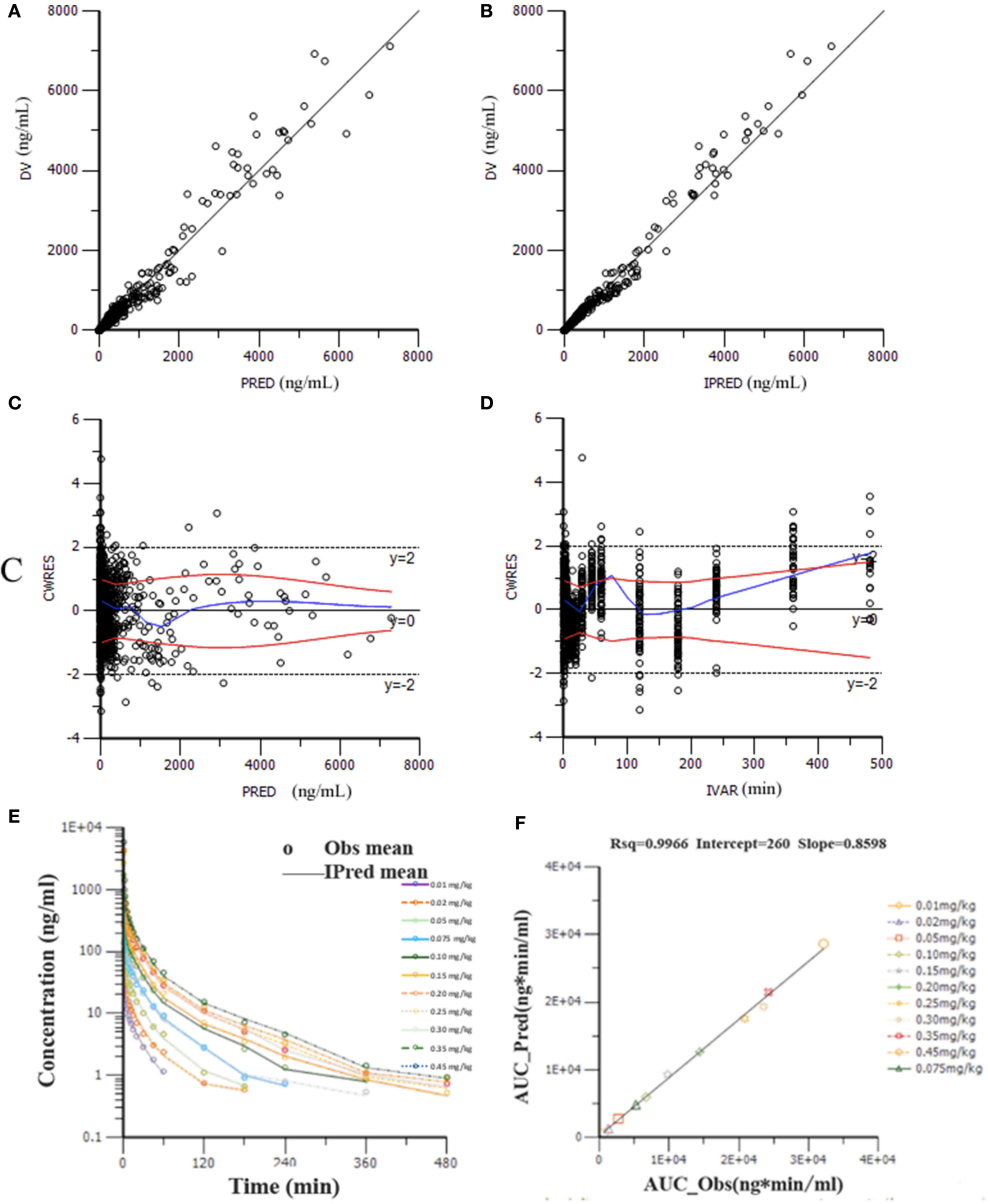
Goodness-of-fit plots for the final PK model: the population (PRED, **A**) or individual (IPRED, **B**) predicted vs. individual observed plasma concentrations (DV); The conditionally weighted residuals (CWRES) against population predictions **(C)** and time **(D)**; The observed and individual predicted mean plasma concentrations against time **(E)**, observations are marked as “°”; Relationship of elimination exposure, calculated by non-compartmental methods to that estimated by the final PK model, of subjects to HR7056 after IV administration to healthy volunteers at various doses **(F)**.

Goodness-of-fit plots for the HR7056 final PK model confirmed the quality of its performance: plots of the population or individual predicted vs. individual observed plasma concentrations lie close to the line of *y* = *x*, the conditionally weighted residuals (CWRES) are symmetrically distributed about zero. Whereas, there is some positive bias among the concentrations observed at the later time points. The individual predicted value simulated by the PK model was close to the observed value obtained by the actual clinical trial. The calculated mean systemic exposure of each dose group to HR7056 was similar to the results from non-compartmental analysis. The final PK model was adequately developed and the predictive performance was sufficient to characterize our observations. These are all shown in Figure 2.

### Population pharmacodynamic modeling for BIS

Pharmacodynamic modeling based on the parameters from the final pharmacokinetic models of HR7056, for both the BIS and MOAA/S data, were undertaken. The BIS observations were continuous data and there was a certain lag relationship between BIS and concentration of HR7056, which is shown in Figure S2. Therefore, a sigmoid inhibitory effect model was used to fit the data. In addition, inclusion of age, weight, height, or gender did not result in a significant decrease in the OFV. Therefore, no covariates were included in the final model.

**Figure 3:**
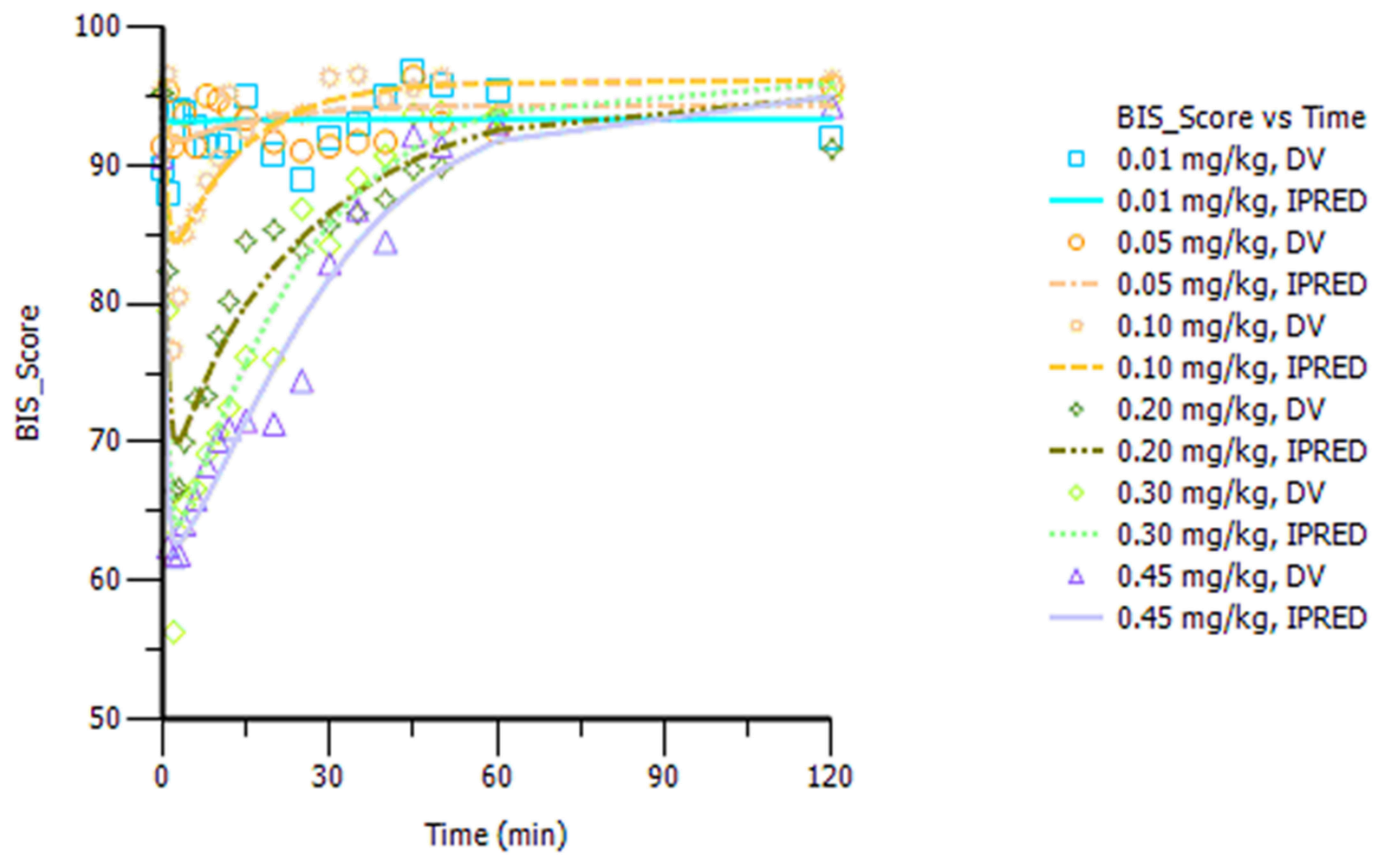
Plot of the observed and individual predicted BIS against time, observations are marked as “DV,” predictions are marked as “IPRED”.

The final model parameters are described in Table 2. Goodness-of-fit plots, such as population or individual predictions vs. observations and CWRES vs. time or population predictions, are shown in Figure S3. Figure S4 shows the visual predictive check for the BIS model, in which tendency of the 95th percentiles of the predictions are not in accordance with that of the observations and this will be commented on further in the section Discussion. The individual predicted and observed BIS of six dose groups (low, median, and high dose) are well-fitted through the sample time (shown in Figure 3). Overall, these figures demonstrated that the presented model adequately described observed changes in BIS during and after HR7056 administration and that all parameters of the model were estimated with acceptable precision.

**Table 2:** Summary of final population pharmacodynamic models of HR7056.

**Parameter**	**Typical value (RSE%)**	**IIV (RSE%)**
**FINAL BIS MODEL**		
K_e0_ (min^−1^)	0.0855 (7.4)	51.2 (25.1)
IC_50_(ng mL^−1^)	503 (10.3)	41.1 (20.2)
Hill coefficient (γ)	1.50 (7.8)	86.4 (36.5)
E_0_	95.3 (0.4)	1.56 (22.1)
I_max_	47.9 (7.4)	15.6 (30.1)
σ	0.0653 (2.2)	–
**FINAL MOAA/S MODEL**		
B1	−8.52 (13.8)	–
B2	−2.44 (10.5)	–
B3	−1.30 (13.0)	–
B4	−1.15 (16.8)	–
B5	−0.895 (21.6)	–
K_e0_ (min^−1^)	0.05 (7.3)	38.3 (11.0)
IC_50_ (ng mL^−1^)	436 (15.6)	45.5 (29.8)
Hill coefficient (γ)	1.50 (14.6)	55.6 (40.1)
I_max_	27.9 (9.4)	17.6 (32.6)

*B1~B5, Baseline value; –, Not applicable; RSE%, Percentage relative standard error; IIV, Inter-individual variability*.

### Population pharmacodynamic modeling for MOAA/S

The categorical MOAA/S data were modeled by means of ordered logistic regression. The drug acts to increase the baseline logit according to a sigmoid E_max_ model based on the predicted effect-site concentration (C_e_). The logits were back-transformed to cumulative probabilities using the inverse of the logit transformation. Subsequently, the probabilities for each category were calculated by subtraction from the cumulative probabilities, with the probability to observe a MOAA/S score ≤ 5 being 1. The MOAA/S models were initially explored using the pharmacokinetic parameters from the final pharmacokinetic model of HR7056. Covariate analysis of the effects of gender and age on parameters had been undertaken for HR7056. However, no covariate effects were statistically significant at the 0.01 level. The final model parameters and associated standard errors are shown in Table 2, which shows that all parameters of the model have been estimated with acceptable precision. A visual predictive check for the final model is shown in Figure S5. The median of the observed frequencies basically falls within the 95% confidence interval of the predicted value produced by the simulation, except for the MOAA/S score of 1 and 5. Figure S6 shows the predicted probability of MOAA/S score changing over time of different dose regimens (0.01–0.45 mg kg^−1^). From these plots, we can see that all subjects are fully alert (MOAA/S score of 5) when dose ≤ 0.05 mg kg^−1^; Starting from a dose of ≥0.075 mg kg^−1^, the subjects' MOAA/S score was reduced to 4–0 after dosing and the probability of progression to lower scores and the time of returning to baseline levels (MOAA/S score of 5) increased with dose; when the dose ≥0.30 mg kg^−1^, almost all subjects' MOAA/S score can be reduced to 2 or less. These predictions are in line with the observations. Overall, these diagnostics show that our final model is adequately developed and that the predictive performance is sufficient to characterize our observations.

### Simulations

**Figure 4:**
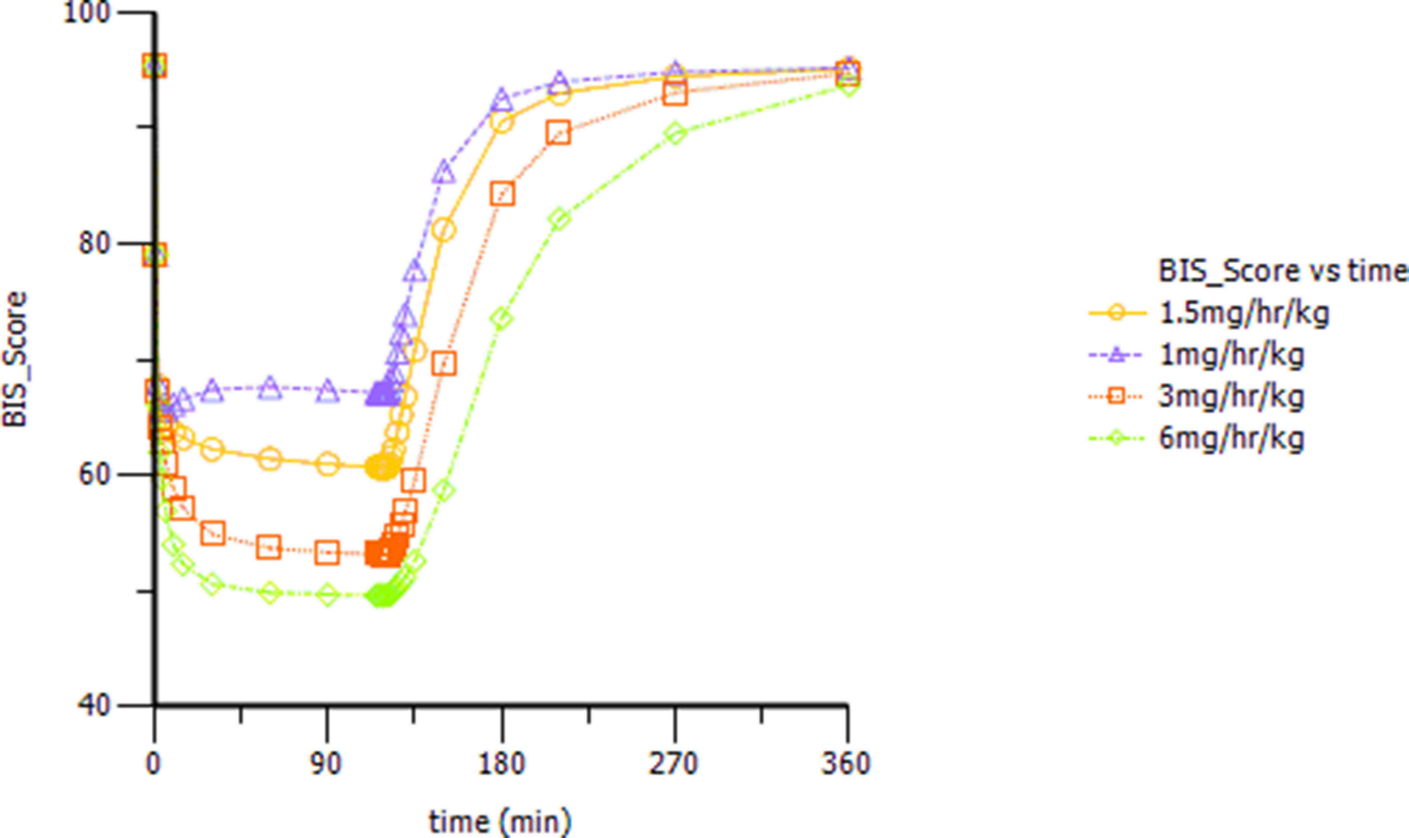
Monte-Carlo simulations of BIS score changing over time during and after a 0.4 mg kg^−1^ min^−1^ (infusion within 1 min) initial loading dose of HR7056 followed by 1.0, 1.5, 3.0, and 6.0 mg kg^−1^ h^−1^ (infusion for 2 h) maintenance doses, respectively.

**Figure 5:**
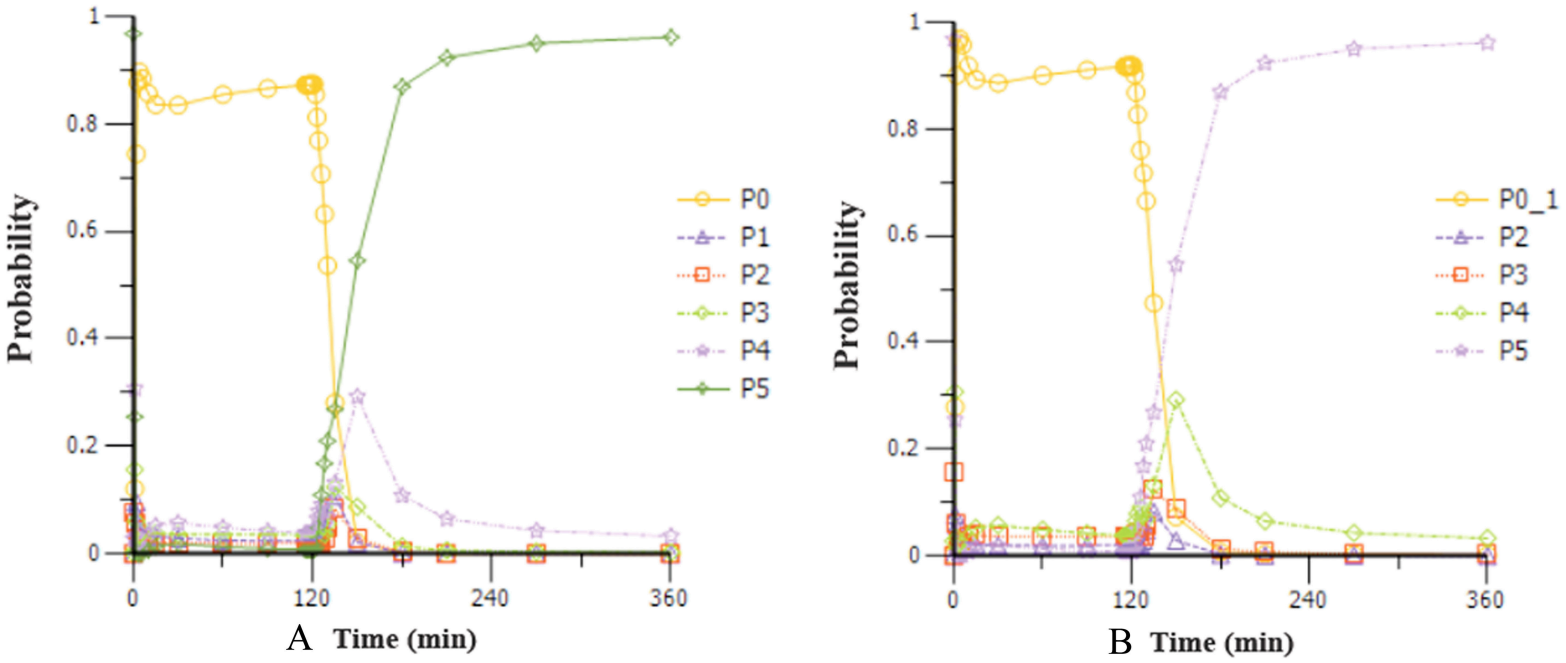
Monte-Carlo simulations of the probability of MOAA/S score changing over time during and after a 0.4 mg kg^−1^ min^−1^ (infusion within 1 min) initial loading dose of HR7056 followed by 1.5 mg kg^−1^ h^−1^ (infusion for 2 h) maintenance dose. [PX represents the probability of MOAA/S score = X; P0_1 means the probability of MOAA/S < 2; Compared to **(A)**, P0_1 in **(B)** is the sum of P0+P1, which means the probability of MOAA/S < 2].

Based on the observations, we chose a loading dose of 0.4 mg kg^−1^ min^−1^ (infusion within 1 min) so as to reach the target effect for induction of anesthesia quickly. Then, simulations of the loading dose with different maintenance dose regimens were performed. The optimal regimen, in terms of achieving BIS values between 60 and 65, and MOAA/S < 2 for more than 1.5 h so as to allow for the induction and maintenance of anesthesia, seemed to be a 0.4 mg kg^−1^ min^−1^ initial loading dose followed by 1.5 mg kg^−1^ h^−1^ maintenance doses. Figure 4 shows the Monte-Carlo simulations of BIS score changing over time of different dose regimens using the final PK/PD model. It can be seen from the figure that the BIS score can be maintained within 60–65 by the administration regimen of loading dose (0.4 mg kg^−1^ min^−1^, infusion for 1 min) + maintenance dose (1.5 mg kg^−1^ h^−1^, infusion for 2 h) and last longer than 1.5 h. Figure 5 shows the Monte-Carlo simulations of the predicted probability of MOAA/S score changing over time of the above dose regimen. Sedation was rapid with all subjects, and about 90% of the subjects reached a MOAA/S score of zero during the first 10 min of administration under this dose regimen (as shown by yellow circles line in Figure 5A). Meanwhile, MOAA/S score < 2 was predicted in almost 100% of the subjects (Figure 5B, yellow circles line P0_1), and with the maintenance dose infusion, the probability of MOAA/S score < 2 was maintaining above 90%.

## Discussion

We developed the PK/PD model that characterizes the relationship between HR7056 arterial plasma concentrations and the changes in BIS and MOAA/S. This is the first report of the PKs and PDs of HR7056 in Chinese healthy population. A three-compartment model fit the PK data well. We did not make attempts to model the PKs and PDs concurrently because the quality of the kinetic data was so reliable that a two-stage approach was used. Firstly, the pharmacokinetic parameters for each subject were estimated, using the final PK compartmental model, which was then used to simulate arterial concentration profiles for the pharmacodynamic modeling. The present study suggests that the high clearance of HR7056 is responsible for its short duration of action. In healthy subjects, the total elimination clearance was 1.49 L min^−1^. While, in the study of Wiltshire et al. (2012), the total elimination clearance is a bit lower (1.11 L min^−1^). We speculate that this may be related to differences in activity of non-specific tissue esterases between Caucasians and Chinese subjects. However, the total volume of distribution in our study (35.3 L) is similar to that in the study of Wiltshire (34.9 L).

Since strict entry criteria was set for the clinical trial, the variation of demographic characteristics of the healthy subjects was small. Inclusion of age, weight, height, or gender in the population PK model did not result in a significant decrease in the OFV. No covariate effects were observed for the pharmacokinetic parameters of HR7056.

Sigmoid inhibitory effect pharmacodynamic models were successfully fitted to both BIS and MOAA/S data. The BIS observations were continuous data and a sigmoid inhibitory effect model was directly used to fit the data. Also, we found no covariate effects for pharmacodynamic parameters. The changes in plasma HR7056 concentrations were reflected in BIS, with an estimated half-life of 8.1 min. Half of the maximal effect was attained at 503 ng mL^−1^. The individual predictions resulting from the final BIS model fitted the observed data well. However, the visual predictive check for the BIS model showed that the present model did not describe observed changes in BIS of high score adequately. The baseline level of the BIS observation is 100, indicating no sedation effect. Whereas, the simulated prediction presented a BIS higher than 100, which can be ameliorated with a certain degree by changing the error model. The difference between prediction and observation is presumed to be caused by residual random effects.

The basis for MOAA/S model is a sigmoid E_max_ model, using the logit of cumulative probabilities of MOAA/S scores rather than the MOAA/S scores themselves. The equilibration between plasma concentrations and effect-site concentrations for HR7056 is fairly slow, with an estimated half-life for effect-site equilibration of 13.9 min. Subjects achieved half of the maximal MOAA/S effect at an effect-site concentration of 436 ng mL^−1^. The predicted probability of MOAA/S score changing over time of different dose regimens are in line with the observations. According to the visual predictive check for the final model, we can see that, as compared to the other MOAA/S categories, the 90% prediction interval obtained through simulation for MOAA/S of 1 and MOAA/S of 5 are both relatively wider, exceeding 30%. In the meanwhile, the median of the observed values does not fall completely within the 95% confidence interval obtained through simulation. However, trend of the confidence interval generated by the simulation is consistent with that of the observations, and the depth and inflection point of the trend are also consistent.

Overall, HR7056 showed dose-related sedative effects in human subjects, which are the same with the study of Antonik et al. (2012). At a dose of ≤ 0.05 mg kg^−1^, the subject's BIS and MOAA/S were hardly affected by the drug; when the dose was ≥0.075 mg kg^−1^, sedation occurred within 2 min after injection and the peak of sedation could be observed within 4 min. The maximum decrease of BIS and the time to return to baseline after the fall were increased with the dose ascending. These pharmacodynamic characteristics were similar to those of remimazolam reported by Antonik et al. (2012).^.^However, in the dose range of 0.1–0.35 mg kg^−1^, which was equivalent to Remimazolam of 0.075–0.3 mg kg^−1^ after labeling dose conversion, a considerably quicker recovery was observed in Chinese subjects treated with HR7056 (molecular weight 611.5 g mol^−1^) than with remimazolam (0.075 ~ 0.3 mg kg^−1^, molecular weight 439.3 g mol^−1)^ in Antonik's study at the same dose level, with a median time for return to fully alert ranging from 0 to 21.5 min for HR7056 (shown in Table S2) in comparison with 5.5–31.5 min for remimazolam. This may be related to the higher clearance of HR7056 in Chinese subjects. In addition, the sedative onset and sedation recovery time of HR7056 and remimazolam were both shorter than those of midazolam and the sedation depths of two drugs were both deeper than that of midazolam.

The intravenous pumping trial of HR7056 was designed to study the effects of the induction and maintenance of anesthesia after administration of HR7056 using a loading dose followed by maintenance dose. For studies of continuous dosing, we should firstly identify the dose of HR7056 that could achieve the target effect, which was loss of consciousness, defined as MOAA/S score < 2 for at least 1.5 h. In our single-dose study, the median plasma concentration of HR7056 for MOAA/S < 2 was 556 ng mL^−1^ with a 70% quantile of 820 ng mL^−1^. The median BIS score for MOAA/S < 2 was 62 and the 70% quantile was 65. Based on this, preliminary Monte-Carlo simulations was performed. We set the target value for BIS to 65 and the target concentration for HR7056 to 820 ng mL^−1^. Using the final PK/PD model, combined with the results of single-dose study, the dosing regimen was finally set as: HR7056 loading dose 0.4 mg kg^−1^ min^−1^ (infusion within 1 min) followed by 1.5 mg kg^−1^ h^−1^ maintenance doses (infusion within 2 h). Then, BIS score can be maintained within 60–65, and MOAA/S scores were expected to be <2.

In conclusion, we present a PK/PD model that adequately describes the sedative effects of HR7056 in Chinese healthy volunteers. No covariate effects considered to be clinically relevant were observed. Nevertheless, the limited number of subjects in our study and the strict entry criteria set for the clinical trial could have obscured the covariate effect. Therefore, it cannot be concluded from these fairly limited data and narrow demographic characteristics that which covariate will be clinically relevant to the PK/PD of HR7056, but the possibility of the covariate effect should be borne in mind in future studies, which will have more data of target population added in. In the meanwhile, simulations based on the final models can predict the target effect of the induction and maintenance of anesthesia and guide dosing optimization, which will enable maintenance doses to be given more accurately for future study.

## Author contributions

YZ wrote the manuscript. PH, XC, and JJ designed the research. YZ, PH, HW, and JJ performed the research. XC, YH, NS, and KS organized the clinical trial. YZ and JW analyzed the data.

### Conflict of interest statement

The authors declare that the research was conducted in the absence of any commercial or financial relationships that could be construed as a potential conflict of interest.
