# Corrigendum: Population Pharmacokinetic/Pharmacodynamic Model-Guided Dosing Optimization of a Novel Sedative HR7056 in Chinese Healthy Subjects

**DOI:** 10.3389/fphar.2019.00251

**Published:** 2019-03-19

**Authors:** Ying Zhou, Pei Hu, Yuguang Huang, Nuoer Sang, Kaicheng Song, Hongyun Wang, Jinhua Wen, Ji Jiang, Xia Chen

**Affiliations:** ^1^Clinical Pharmacology Research Center, Peking Union Medical College Hospital, Peking Union Medical College and Chinese Academy of Medical Sciences, Beijing, China; ^2^Department of Pharmacy, The First Affiliated Hospital of Nanchang University, Nanchang, China; ^3^Department of Anesthesiology, Peking Union Medical College Hospital, Peking Union Medical College and Chinese Academy of Medical Sciences, Beijing, China; ^4^Clinical Trial Center, China National Clinical Research Center for Neurological Diseases, Beijing Tiantan Hospital, Capital Medical University, Beijing, China

**Keywords:** population pharmacodynamics, population pharmacokinetics, sedation, modeling, benzodiazepine

In the original article, we neglected to include the funder “Chinese National Natural Fund grant” (No. 81671369), to XC.

An author name was also mistakenly incorrectly spelled as “Bei Hu.” The correct spelling is “Pei Hu.”

Additionally, there was an error regarding the affiliation for Ying Zhou. As well as having affiliation “Department of Pharmacy, The First Affiliated Hospital of Nanchang University, Nanchang, China,” she should also have “Clinical Pharmacology Research Center, Peking Union Medical College Hospital, Peking Union Medical College and Chinese Academy of Medical Sciences, Beijing, China.”

Lastly, Xia Chen, Yuguang Huang, Nuoer Sang, and Kaicheng Song were not included as authors in the published article. They have all contributed significantly to this manuscript by organizing the clinical trial, which we carelessly overlooked.

The corrected **Author Contributions Statement** appears below.

“YZ wrote the manuscript. PH, XC, and JJ designed the research. YZ, PH, HW, and JJ performed the research. XC, YH, NS, and KS organized the clinical trial. YZ and JW analyzed the data.”

The authors apologize for these errors and state that they do not change the scientific conclusions of the article in any way. The original article has been updated.

