# Supplementary material for: Population Pharmacokinetic/Pharmacodynamic Model-Guided Dosing Optimization of a Novel Sedative HR7056 in Chinese Healthy Subjects
Source: Front Pharmacol. 2018 Nov 19;9:1316. doi: 10.3389/fphar.2018.01316 (PMC6252322; doi:10.3389/fphar.2018.01316)

Table S1 Modified Observer’s Assessment of Alertness/Sedation (MOAA/S) scoring standard

| Score | Responsiveness |
| --- | --- |
| 5 | Fully alert, responds readily to name spoken in normal tone |
| 4 | Lethargic response to name spoken in normal tone |
| 3 | Responds only after name is called loudly and/or repeatedly |
| 2 | Responds only after mild prodding or shaking |
| 1 | Responds only after painful trapezius squeeze |
| 0 | Does not respond to painful trapezius squeeze |

Table S2 Modified Observer’s Assessment of Alertness/Sedation (MOAA/S)—Time to Fully Alert

|  | Time to Fully Alert (min) | | | | | | | | | | | | |
| --- | --- | --- | --- | --- | --- | --- | --- | --- | --- | --- | --- | --- | --- |
|  | Midazolam  (mg/kg ) | | HR7056  (mg/kg ) | | | | | | | | | | |
|  | 0.075 | 0.12 | 0.01 | 0.02 | 0.05 | 0.075 | 0.10 | 0.15 | 0.20 | 0.25 | 0.30 | 0.35 | 0.45 |
| N | 8 | 8 | 3 | 3 | 3 | 6 | 6 | 6 | 6 | 6 | 6 | 9 | 9 |
| Minimum | 3.0 | 19.0 | 0.0 | 0.0 | 0.0 | 0.0 | 0.0 | 7.0 | 9.0 | 7.0 | 11.0 | 9.0 | 19.0 |
| Median | 36.5 | 54.0 | 0.0 | 0.0 | 0.0 | 0.0 | 0.0 | 12.5 | 10.0 | 21.5 | 19.0 | 19.0 | 29.0 |
| Maximum | 119.0 | 119.0 | 0.0 | 0.0 | 0.0 | 11.0 | 9.0 | 24.0 | 39.0 | 29.0 | 29.0 | 29.0 | 44.0 |

Time to fully alert was measured from the time of the first postdose MOAA/S score of <5 to the first of 3 consecutive MOAA/S scores of 5.

If a subject never became sedated, time to fully alert was set to 0.

**Figure S1** Chemical structures of remimazolam Tosilate (HR7056).

**Figure S2** Plots of the certain lag relationship between BIS and concentration of HR7056.


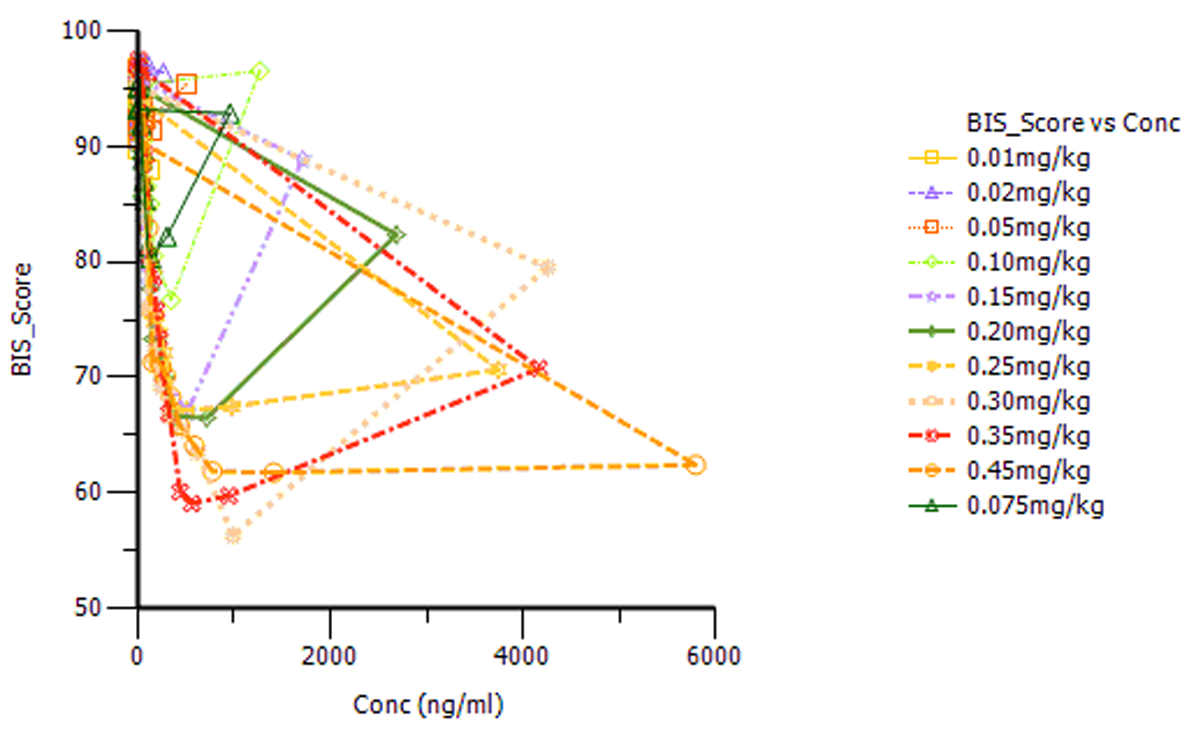


**Figure S3** Goodness-of-fit plots for the final PD model for BIS: the population (PRED, A) or individual (IPRED, B) predicted vs individual observed BIS (DV); The conditionally weighted residuals (CWRES) against population predictions (C) and time (D).


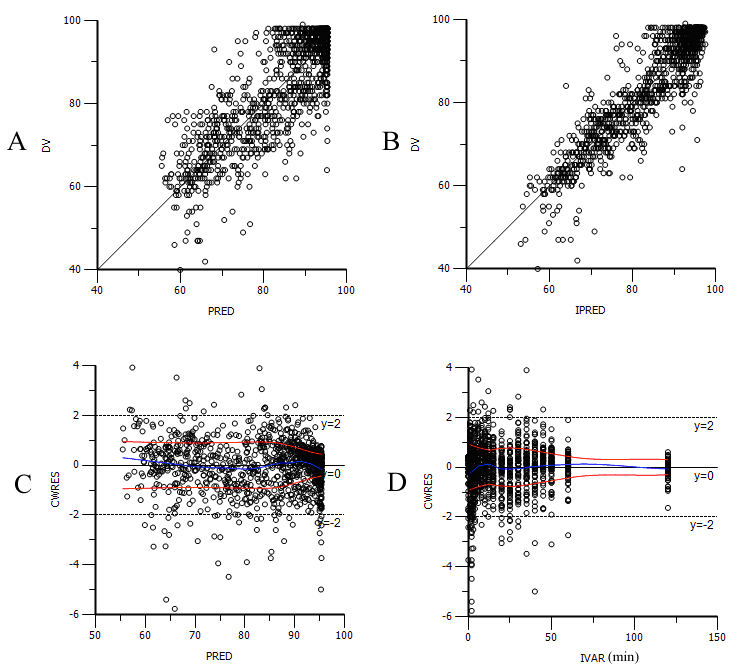


**Figure S4** A visual predictive check for the BIS model. The black lines show the median (solid line) and 5th and 95th percentiles (dashed lines) of the observed BIS. The shaded areas denote the 95% confidence intervals around the percentiles.


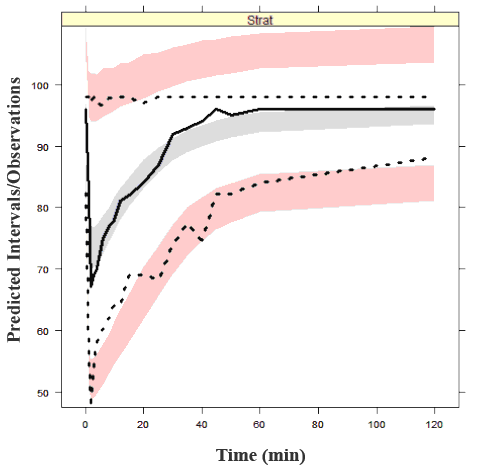


**Figure S5** A visual predictive check for the MOAA/S model. The median of the observed frequencies is shown with blue lines, which basically falls within the 95% confidence interval of the predicted value produced by the simulation (shaded areas), except for the MOAA/S score of 1 and 5. The VPC were truncated at 85 min after the start of the study, because the lower data density beyond this point had a confounding influence on the VPC. (2.5<DV<=3.5 represents MOAA/S score of 3; the rest may be deduced by analogy)


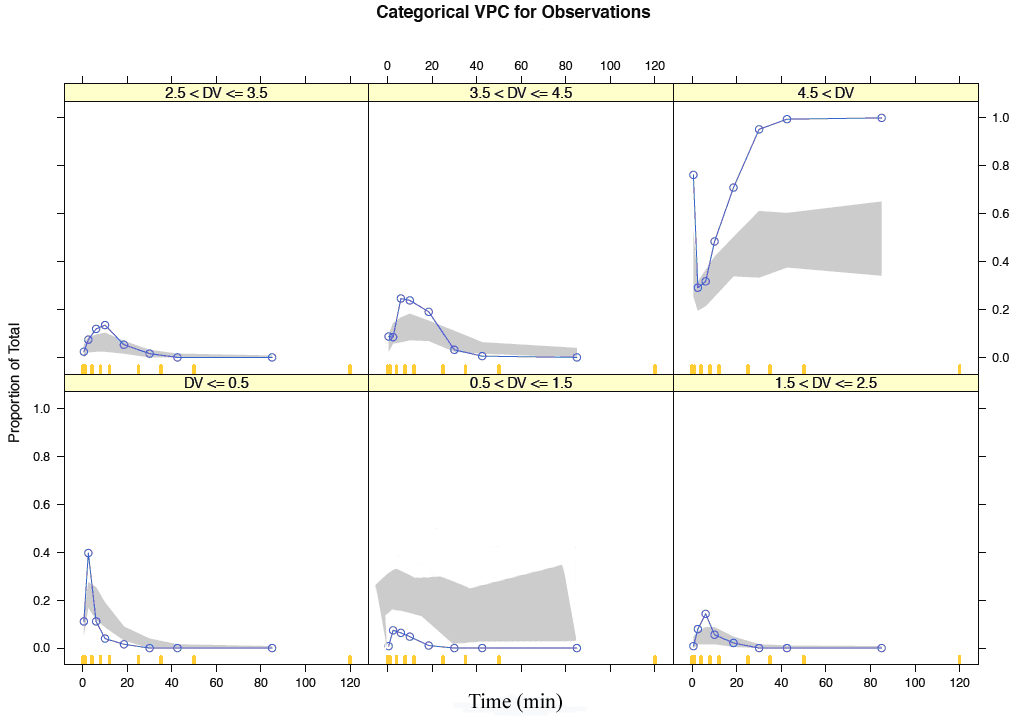


**Figure S6** Plots of the predicted probability of MOAA/S score changing over time of different doses (0.01-0.45 mg kg^-1^). (PX represents the probability of MOAA/S score = X)


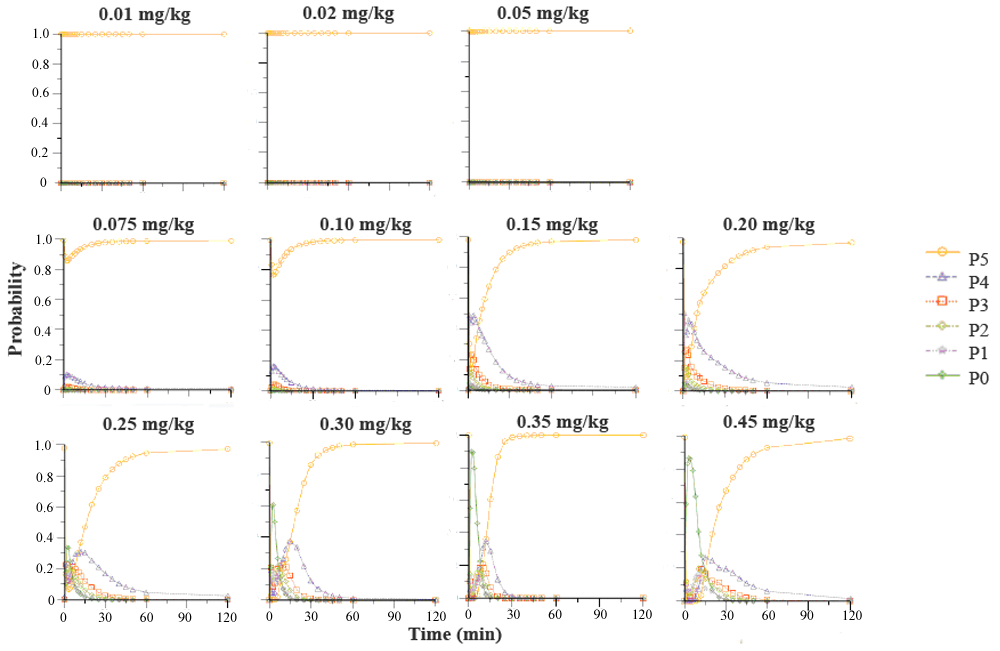

Supplement: Supplementary file 1 [file Table_1.DOC]
